# Influence of Prenatal Methamphetamine Abuse on the Brain

**DOI:** 10.3390/epigenomes4030014

**Published:** 2020-07-14

**Authors:** Anežka Tomášková, Romana Šlamberová, Marie Černá

**Affiliations:** 1 Department of Medical Genetics, Third Faculty of Medicine, Charles University, 100 00 Prague, Czech Republic; anezka.tomaskova@lf3.cuni.cz; 2 Department of Physiology, Third Faculty of Medicine, Charles University, 100 00 Prague, Czech Republic; romana.slamberova@lf3.cuni.cz

**Keywords:** methamphetamine, prenatal, drug addiction, striatum, prefrontal cortex, hippocampus, dopamine, serotonin

## Abstract

Methamphetamine (MA), a psychostimulant, has become a serious problem in recent years. It is one of the most widely abused psychostimulants in the world. In the Czech Republic, ecstasy is the most commonly used non-cannabis drug, followed by hallucinogenic fungi, LSD, MA, cocaine, and finally heroin. The prevalence of the usage of all addictive substances is highest in the age category of 15–34. Approximately 17.2% of registered drug addicts, both male and female, in the Czech Republic use MA as their first-choice drug. This group consists mostly of women who are unemployed and addicted to MA (85%). Almost half of the addicted women switched to MA from other drugs in the course of pregnancy. Psychostimulants such as amphetamine and its synthetic derivate MA induce feelings of calm and happiness by suppressing anxiety and depression. When MA is abused for longer periods, it mimics symptoms of mania and can lead to the development of psychosis. MA is often abused for its anorectic effect, its simple preparation, and compared to heroin and cocaine, its low price. There are significant differences in the susceptibility of users to the stimulant, with reactions to MA fluctuating from person to person. Molecular mechanisms related to the variable response among users might represent an explanation for increased addiction-associated bipolar disorder and psychosis. Currently, there is limited information regarding genetic mechanisms linked to these disorders and the transmission of drug addiction. As such, animal models of drug addiction represent significant sources of information and assets in the research of these issues. The aim of this review is to summarize the mechanism of action of methamphetamine and its effect on pregnant addicted women and their children, including a detailed description of the anatomical structures involved.

## 1. Introduction/Background

### 1.1. Drug Dependence

**Figure 1:**
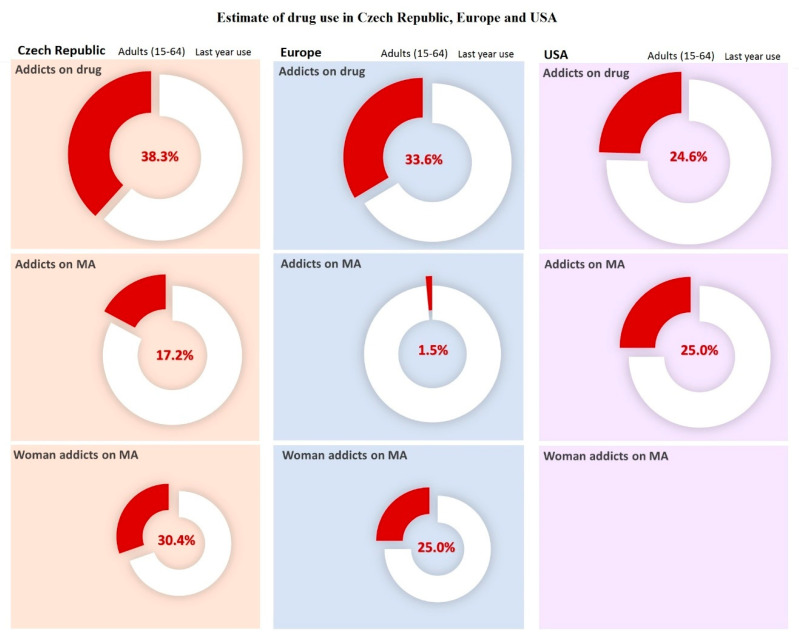
Estimates of drug use in Czech Republic, Europe, and USA. Data was carried out in selected countries over a year in 2017. For American female addicts of methamphetamine (MA), data are not available. For the Czech Republic, the population was estimated at 10,665,677 people for the year 2018 according to United Nations data. Data adopted and modified according to National Monitoring Center for Drugs and Addiction, Annual Report [2]. For Europe, the population was estimated at 513,000,000 people for year 2018 according to Eurostat. Data taken over and modified by The European Monitoring Center for Drugs and Drug Addiction [3]. For USA, the population was estimated at 327,200,000 people for the year 2018, according to United Nations data. Data taken over and adjusted by the National Institute on Drug Abuse [4].

Addiction is a set of behavioral, cognitive, and physiological conditions. The main cause for its development is the repeated use of addictive substances; typical symptoms include a strong preference for using the substance repeatedly, altered consciousness after use, and persistent usage despite the damaging effects. Additionally, use of the drug takes priority over other activities and commitments, there is a gradual increase in tolerance, and somatic withdrawal sometimes occurs when use of the substance stops. A frequent motive for substance abuse is curiosity, which can subsequently lead to a state of addiction. Many users first try the drug in order to find out “what’s it like” [1]. Other possible motives include stress or problems that the individual is incapable of coping with in any other way, leading to drug use as a means to escape reality. Drug addiction is a global problem. It directly affects a large part of society, and its indirect effects on the families and surroundings of users are even more extensive (Figure 1).

In reducing drug usage, prevention is an important factor. In the prevention of addiction and the use of alcohol and non-alcoholic drugs, the World Health Organization (WHO) distinguishes three targets: (1) primary prevention, i.e., preventing drug use in those who have not had direct contact with the drug; (2) secondary prevention, i.e., preventing addiction in drug users; and (3) tertiary prevention, i.e., preventing the serious and lasting health and social problems created by using drugs, including both resocialization or social rehabilitation and measures aimed at decreasing the transmission of infectious diseases during intravenous drug application (e.g., harm reduction programs) [5].

The initial phase of drug addiction, i.e., when the user is experiencing euphoria and is still discovering the drug’s effects, is called the phase of experimental use. During the phase of social use, the user begins to take the drug more regularly, often at higher doses; during this phase, addiction begins to show and the drug slowly becomes a source of various problems. In the course of everyday usage, the drug begins to impact such things as daily routines, moral values, employment, and friendships. Ultimately, the user loses control of their addiction [6]. Chemical dependency (i.e., the need for more of a substance to get the same effects) marks the last stage of addiction, often ending in a fatal overdose or total organ failure.

The treatment of drug addiction is a very complicated and complex process, since addiction affects not only the physiology but also the psychology of the affected individual, often requiring the complete removal of the user from their social environment [6,7,8]. The final goal of treatment is to completely eliminate the addiction. Treatment progresses gradually, since overly quick withdrawal can produce unwanted side-effects, including potentially health-threatening complications [9].

### 1.2. Stimulant Drugs: Characteristics and the General Mechanism of its Effect

This paper will focus on the effects of methamphetamine (MA), which is considered a stimulant. Stimulant drugs can be further specified as psychostimulants, psychoanaleptics, psychomimetics, and psychomotor stimulants [9]. Caffeine can be undoubtedly be considered the most famous stimulant, whereas amphetamines represent a significant group of illegal stimulants. Other examples include cocaine and its subsequent product, “crack”, as well as “party drugs”, which are derived from amphetamines [9]. Psychostimulants have strong stimulant effects, affecting not only the central nervous system (CNS), but also the organism. The typical effects of this group of substances include increased wakefulness, shorten sleep cycles, suppressed fatigue, accelerated thinking, improved association and memory, euphoria, and a pleasant feeling of both mental and physical strength [10]. The stimulant effect also leads to increased blood pressure and pulse rate (potentially inducing a hypertensive crisis), changes in blood distribution in favor of muscle tissue, and increased muscle tone. Additionally, there is bronchodilation, which leads to shallow breathing [8,10].

The mechanism of these effects is based on direct interactions with neurons and the information transmitted between them. The mechanisms differ among specific substances, yet the basic principle remains the same, i.e., the substances in question increase the mediator concentration in synapses between neurons, with the effect of altered signal transmission. MA, for example, has a destructive influence on dopamine nerves and their endings. Due to adaptive cellular mechanisms, tolerance and addiction develop after repeated usage.

Several mechanisms participate in the development of addiction. Neuroadaptations, which are changes in the organism directed at maintaining homeostasis, is one of them. After the drug effects fade, withdrawal syndrome appears, with typical symptoms including generalized exhaustion, intense fatigue, and widespread body pain. Frequent mood changes and depression are also present. This state usually subsides relatively quickly; however, in some cases, it can persist for several days. Psychostimulants can also lead to anxiety, increased nervousness, and depression. Even a single use can potentially induce a panic attack [11]. Long-term usage can lead to psychosis, with states of paranoia, feelings of persecution, and feeling of being under threat [12]. These problems develop after ingestion of a psychostimulant and cannot be simply explained by the intoxication, yet are not a part of drug cessation states either. Ceasing drug usage is not guaranteed to resolve these problems [13].

## 2. Methamphetamine

Methamphetamine (also pervitin, methylamphetamine, desoxyephedrine, and methedrine) belongs to the group of wake-promoting amines [14]. The first discovered member of this group, amphetamine, was synthesized in 1887. The group of wake-promoting amines includes hundreds of substances, which were (and still are) often used as a treatment for exhaustion, narcolepsy, increased appetite, and sometimes abused by the military in order to increase the performance of combat units. The base substance for MA synthesis is ephedrine, along with lye and red phosphorus used in its manufacture. A minimum level of knowledge of at least high school grade is generally required, since when done imperfectly this may threaten the health of the user [9]. Pure MA can be obtained in crystalline form; it is, however, more common to be sold as a microcrystalline white powder with a bitter taste and without a perceptible aroma. Due to the presence of additives originating from home manufacturing, the powder often has a yellow or purple discoloration [15].

MA can be classified as a non-catecholamine sympathomimetic substance, showing a sympathomimetic effect even without possessing the catecholamine structure [14]. MA exhibits stimulatory effects on the CNS [16,17,18] by releasing noradrenaline (NA) from noradrenergic neurons that act on alpha receptors [19]. Amphetamines, on the other hand, have little influence on these receptors. Both serotonin (5-hydroxytryptamine; 5-HT) and dopamine are also released after the administration of MA, leading to increased psychomotor activity. MA initially induces alertness, suppresses fatigue, and increases energy; with higher doses, it produces feelings of euphoria, blissfulness, and increased self-confidence [20,21].

MA accelerates heart activity and increases blood pressure, which leads to hyperthermia, bronchial dilation, and pupil dilation [22]. A strong psychological addiction is created with MA, but without physical addiction [23]. MA is well absorbed by the gastrointestinal tract and mucous membranes. Its lipophilic character enables it to pass through the blood–brain barrier with greater ease compared to amphetamine [24]. Once in the CNS, MA causes an increase in monoamine mediator (dopamine, noradrenaline, serotonin) concentrations in the synapses and in the cytosols of neurons. Simultaneously, neurotransmitter reabsorption is inhibited and the degradation of MA is reduced [25]. In high doses, monoamine oxidase (MAO) is also inhibited. Drug inactivation via MAO is prevented due to the presence of a methyl group on the alpha carbon [24]. Benefiting from this protection, MA is widely distributed throughout the body, and its biological half-life is 12 to 34 h [26].

After the effects subside, there is a lack of neuromodulators, leading to an unpleasant state, which is often called withdrawal syndrome. During long-term use, irreversible changes appear in the mitochondrial metabolism, potentially leading to apoptosis of the damaged neuron [25]. Sexual activity is also affected by long-term use. Other effects include increased nervousness, restlessness, insomnia, bruxism, reduced appetite, and subsequent weight loss [27]. Restlessness and paranoia may potentially lead to paranoid psychosis, which can recur even after discontinuation of drug use [28].

Research has determined that different animal species metabolize MA differently. Rats, for example, use aromatic hydroxylation during its metabolism, whereas rabbits use deamination [24,29]. In humans, a substantial amount of MA is excreted via the urine in an unaltered form (approximately 45% within 24 h). Excretion is highly dependent on pH; while up to 76% can be excreted in acidic urine, only about 2% can be excreted in alkaline urine [30,31]. MA metabolites are also excreted in urine—approximately 15% is metabolized by hydroxylation in the liver to hydroxymethamphetamine. About 7% is metabolized by N-demethylation to amphetamine, which is further processed to hydroxyamphetamine (2–4%) and norephedrine (2%), and subsequently hydroxynorephedrine (0.3%) and phenylacetone (0.9%), which are metabolized to benzoic acid and hippuric acid, respectively [24,29].

## 3. Influence of Methamphetamine on the CNS and on Selected Parts of the Brain

Every living organism strives for a dynamic balance, called homeostasis. While under the effect of addiction, this balance is threatened by distinct physical and cognitive events, leading to increased stress in the addicted individual [32,33]. Consequently, their behavior is changed; unless the event corresponds to a cognitive depiction based on previous subjective experience, it is accompanied by an increase in excitement, alertness, and cognitive processing [34,35,36,37,38,39]. The interface between the incoming sensory input and the evaluation process consists of limbic brain structures, including the hippocampus and prefrontal cortex, and the structures directly influencing the limbic system, i.e., the striatum [40].

The control areas in the brain send signals that configure the processing of incoming sensory inputs from moment-to-moment [37,41]. Humans possess unique cognitive flexibility. The brain control system consists of functionally diverse areas that are anatomically separated from the remaining systems of neural response processing [41].The ability to perform countless tasks requires control functions that persist in time and that are capable of not only preventing attention diversion, but of responding quickly to unpredictable demands as well [33].

**Figure 2:**
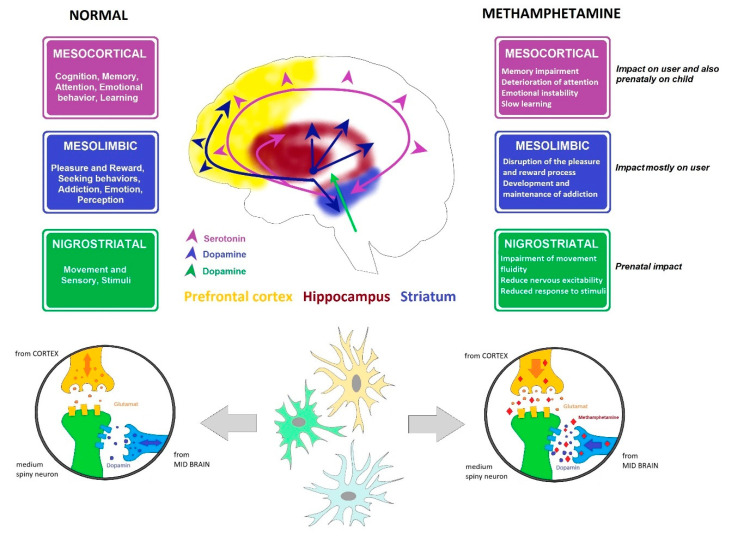
Dopamine and serotonin pathways. Cross-section through the brain showing dopamine and serotonin pathways and their main functions, including affection, mood, memory, sleep, pleasure, reward, and compulsive behavior, and the influence of MA.

The selection of samples from rat brains was based on the fact that MA interferes mostly with dopaminergic and serotonergic neural response pathways. MA enters the terminals or neuron via the monoamine transporters (dopamine transporter, serotonin transporter, or norepinephrine transporter), displaces both vesicular and intracellular monoamines, and facilitates the release of monoamines into the extraneuronal space by synaptic transport in the monoamine transporters. Both pathways (dopaminergic and serotoninergic) connect parts of the brain responsible for motor control, fear, pleasure, reward, and addiction mechanisms (Figure 2) [22,42].

### 3.1. Dopaminergic System

Dopamine is a neurohormone; its release from the hypothalamus inhibits prolactin secretion from adenohypophysis, affects motor system control in the CNS, initiates various behavioral pattern, and modulates the activity of visceral functions. Dopamine is a low molecular weight neurotransmitter and is categorized as a catecholamine. In the bloodstream, dopamine exhibits sympathomimetic effects, such as increasing the systolic blood pressure and the heart rate [43,44]. Dopamine is synthesized from tyrosine or phenylalanine and serves as a precursor for both adrenaline and NA; MAO and catechol-O-methyltransferase (COMT) can degrade it, with homovanillic acid being the final product [45].

Dopaminergic nuclei in the brain are designated as A8, A9, and A10. The most significant are located in the substantia nigra pars compacta (A9) and medially in the ventral tegmental area (A10). Fibers from the substantia nigra are projected into the striatum, and to a lesser extent into the globus pallidus. Fibers from the ventral tegmental area create the mesolimbic dopaminergic system, ending in the ventral striatum, ventral pallidum, septum verum, the amygdala, and the cerebral cortex, mainly the prefrontal cortex and primary motor cortex (Figure 2).

Reduced dopamine concentrations in the striatum causes hypokinesis and muscle tremors; lowering the concentration in the prefrontal cortex leads to memory, attention, and motivation disorders. Using MA increases the amount of dopamine accessible to D3 receptors [46], directly stimulating the reward center in the brain. Repeated administration of MA induces long-lasting deficits in the striatal concentrations of dopamine and its metabolites, tyrosine hydroxylase activity, and dopamine transporter binding sites [17,46].

MA causes degeneration of striatal nerve terminals, as shown by long-lasting depletion of dopamine concentration, dopamine transporter, and vesicular monoamine transporter 2 levels. The ability of MA to mobilize dopamine from intraneuronal stores to the extracellular space via dopamine-transporter-mediated outward transport results in elevated extracellular dopamine concentrations. The neurotoxic effects of MA are postulated to occur from subsequent auto-oxidation of dopamine to highly reactive free radicals [17,22,46]. An alternative model suggests that redistribution of dopamine from vesicular storage pools to the cytoplasmic compartment, allowing intraneuronal oxidation, is the primary cause of dopamine terminal injury [46].

The principle reason lies in the stimulation of the tyrosine kinase receptor (e.g., insulin-like growth factor 1 receptor (IGF-1R)). It is shown to mediate activation of the phosphatidylinositol-3 kinase (PI3K)/Akt signaling pathway, an important mediator of cell survival. PI3K is activated by estrogen, the G-protein-coupled estrogen receptor 1 (GPER1, also known as GPR30). Akt controls expression of the anti-apoptotic molecule Bcl-2 via the cAMP-response element-binding protein (CREB), and can also phosphorylate glycogen synthase kinase 3β (GSK3β) and BAD proteins, thereby inhibiting their pro-apoptotic functions [17,22].

This suggests that the balance among vesicular, cytoplasmic, and extracellular dopamine pools play a key role in the neurotoxic action of MA.

Long-term use leads to a loss of dopaminergic receptors and to reduced dopamine production in general, causing withdrawal states in addicted individuals and triggering the need to increase the dosage of the abused substance [47,48] (Figure 2).

### 3.2. Serotoninergic System

The serotoninergic system in the CNS has a regulatory effect on many functions and modulates the activity of other projection systems. This system is closely linked to the noradrenergic system, which it often supplements. Serotonin is derived from L-tryptophan as 5-HT. Serotonin inactivation is catalyzed through two enzymes, MAO and aldehyde dehydrogenase; the activity of these enzymes leads to the creation of 5–hydroxyindoleacetate (5-hydroxyindoleacetic acid), which is mostly excreted in the urine (as a glucuronic acid conjugate) [49]. Another serotonin metabolic pathway results in the synthesis of melatonin; initially, an acetyl group binds to the amino group of serotonin, resulting in N-acetylserotonin. Subsequently, a methyl group binds the hydroxyl group, creating melatonin.

There are seven known subtypes of serotoninergic receptors (i.e., 5-HT1-7R), both excitatory and inhibitory. The effect of serotonin is strongly dependent on the receptors expressed on the neurons of any given structure. The soma of most neurons in this system are in the raphe nuclei of the reticular formation. Their axons enter both ascending and descending columns, reaching all cortical areas (including the prefrontal cortex) and all limbic system structures (including the hippocampus), with others reaching the striatum, thalamus, hypothalamus, brain stem, cerebellum, and spinal cord. Excessive activity in ascending columns leads to mood changes and behavior disorders. The axons passing through the dorsal horn of the spinal cord are associated with pain transmission (Figure 2).

Reductions in forebrain concentrations of serotonin (5-HT) and its metabolites and a decrease of tryptophan hydroxylase activity after MA administration have been described. Lowered serotonin synthesis causes depression and sleep disorders [17,22,50]. MA also causes hyperthermia, which can be lethal. Release of monoamines into the extraneuronal space by synaptic transport in the monoamine transporters suggests that dopamine receptor activation is crucial for methamphetamine-induced hyperthermia. However, when dopamine pools are empty, serotonin starts to play the main role. MA exerts a hyperthermic effect via dopamine transporters, or via serotonin transporters if the dopamine transporters are absent [22].

### 3.3. The Striatum

The striatum (also called the corpus striatum) is grey matter deep in the telencephalon. It is considered the most significant part of the basal ganglia (evolutionarily one of the oldest structures). The basal ganglia participate in generating and controlling movement, cognitive functions, and the functions of the limbic system.

The basal ganglia are connected to various cerebral pathways; the general signal pathway leads from the cerebral cortex to the basal ganglia. The signal continues from the basal ganglia to the thalamus and finally back to the cerebral cortex. Four types of basal ganglia loops have been described: motor loops, oculomotor loops, limbic circuits, and associative pathways. While describing them, it is important to determine the part of the cerebral cortex from which the stimuli originate; furthermore, it is equally necessary to ascertain the ganglia involved, i.e., functioning as inputs or outputs, and the specific functional area of the cortex that is the intended destination. The information comes predominantly from the motor and somatosensorial areas of the cortex and enters the putamen, taking either direct or indirect pathways. The function of the direct pathway is to support the movement, while the indirect pathway inhibits the thalamus, which increases the excitatory influence on the cortex. Increasing the activity in the direct pathway leads to increased motor activity, while the indirect pathway serves mainly to suppress unwanted movements. The activity in both pathways should be balanced; disturbing this balance leads to hyperkinetic and hypokinetic disorders, respectively [32,51].

The substantia nigra pars compacta, which is rich in dopaminergic neurons, plays a substantial role in the modulation of pathway activity. Dopamine increases the activity of the direct pathway via D1 receptors, while also decreasing indirect pathway activity via D2 receptors ([32,45,52]. The basal ganglia participate in motor function control (and to some extent, cognitive function control as well). Generally, they exert an inhibitory influence on motor activity; motor cortex neurons suppress cortical stimuli both through direct feedback and through the reticular formation. The neurons are activated even before the movement begins, possibly indicating that they take part in its planning as well [52]. It is generally assumed that they also participate in the control mechanisms of intricate movement patterns, such as writing, ball games, and speech [53].

### 3.4. The Prefrontal Cortex

The prefrontal cortex represents one of the largest cortical areas of the human brain, accounting for roughly 29% of its volume, and is responsible for higher functions. The cortex area of the frontal lobe, which does not belong to motor areas, represents a significant part of the association cortex [54]. There are links to the visual, olfactory, and auditory cortices, providing integration of sensory inputs. The prefrontal cortex, which receives information from various sources, is responsible for planning, decision-making, and creating new ideas. It has connections to the entire brain, especially to the rostral thalamus.

It is the only area of the cortex with projections directly into the hypothalamus and septal areas, with a significant role in the regulation of the limbic system. There is a direct bidirectional link between them, with the prefrontal cortex taking part in learning and memory processes [54,55]. Damage to the prefrontal cortex manifests in severe psychological disorders, including apathy, memory loss, aggression, obsession, loss of social restraint, and emotional instability [54,56,57,58,59].

### 3.5. Hippocampus

The hippocampus is a paired structure in the telencephalon, occupying the middle part of the temporal lobe in both hemispheres. Information from the cerebral cortex and limbic system is processed in the hippocampus and subsequently sent to the frontal thalamus, then ultimately back to the cerebral cortex. This rhythmic, repetitive activity of the hippocampus creates a basis for complex integration processes, such as storing of information as long-term memory [60]. It is part of the limbic system, mediating both short-term and long-term memory and orientation in space [61,62,63]. The ability to orientate in space and time allows the individual to perceive their position in relation to their environment, as well as in relation to changes in velocity and positioning in time [61,64]. Any damage can manifest as learning disorders (long-term memory), disruptions to short-term memory, and even complete amnesia [65].

There is a large number of cells encoding the perception of space in the brain; they are located both in the hippocampus and in other structures, such as the cerebellum and cerebral cortex [66]. The structure of the hippocampus is essentially identical in most mammals [41,67,68,69]. If the subject is placed in a confined and defined space, the pyramidal cells in this area of the brain (along with the granule cells in the cerebellum) begin to produce strong excitatory signals [70,71,72,73]. An analysis of the involved cells can be performed using the open arena experiment with free movement or using either the radial or Morris water mazes [74,75]. The cells are less sensitive to the vertical dimension in land-based mammals (e.g., rats), while in bats all three basic dimensions produce strong signals [61,76]. The cells associated with head orientation, found in several brain structures linked to the hippocampus, were discovered by Taube et al. [77]. These cells produce signals when the animal looks in a certain direction; unlike the visual angle, the position in space is not relevant for the function of these cells.

Additionally, cells sensitive to both the head direction and general orientation of the animal in space were also found in the hippocampus [61,76,78]. Any disruption of this structure can lead to the emotional instability of the animal, manifesting as anxiety states, depression, or obsession [56,79,80,81,82].

## 4. Methamphetamine Use by Pregnant Addicted Women

Women exhibit a more sensitive reaction to MA compared to men (e.g., addiction forms faster); however, the response to treatment is better as well, but they are also more susceptible to relapse when abstaining [23,83]. Recently, usage of MA among pregnant women has increased [83,84]. Pregnancy has a significant effect on drug sensitivity and metabolism. Studies have shown increased sensitivity to cocaine, which can cause sudden fetal death, while the sensitivity to methadone is decreased, meaning higher doses are necessary [28,31,85]. According to Vavříková [28], the use of heroin, cocaine, and MA are often accompanied by malnutrition and hyperpyrexia. The intravenous route is common and was found in 60% of women addicted to MA [7,28]. Long-term use of this drug leads to physiological changes, changing both plasma and total body water volumes. During pregnancy, this can significantly affect the MA concentration peak, distribution volume, and the biological half-life [30,31].

The placental metabolism is strongly affected by drug use as well [86]. There are several changes in the placenta—in cellular membranes, in the protein links, and in the molecular weight of body compounds, often preventing proper nutrient transfer, lowering blood (and subsequently oxygen) flow rate, and allowing a higher drug permeability to the fetus [30]. In the uterus, calcification and morphological damage to the placenta can cause miscarriage or maternal death [7,87]. It was shown that MA passes the placental barrier easily, with around 50% of the drug quantity being present in the fetal circulatory system as compared to the maternal system [88,89,90]. MA is metabolized in the liver, and since the fetal liver is not yet able to handle this load the concentration increases steadily over time, surpassing the concentrations found in the maternal system [90].

MA is categorized as a reactive oxygen species (ROS). Despite the difficulties in describing the direct mechanism of teratogenesis during organogenesis, it was sufficiently demonstrated that the levels of most antioxidants are lowered in the embryo during maternal MA use. This results in the absence of an efficient defense against the influence of MA on the organism [91]. Consequences can include oxidative damage to lipids, proteins, or DNA, as well as apoptosis activation and necrotic cell death [92]. Studies have shown that MA decreases the levels of dopamine, NA, and MAO inhibitors, with the subsequent increased synaptic activity having neurotoxic effects on the CNS [34]. Cui et al. believes that the combination of MA with monoamine neurotransmitters can affect fetal brain development [85,93]. This teratogenic agent can cause fetal death during pregnancy and is also capable of causing structural abnormalities, including cleft palate and exencephaly [92,94].

Aside from the direct effect of the drug on the child, it is also necessary to consider the indirect effects. Women addicted to MA are typically unemployed (85%) and unmarried (90%) [28], while also being statistically younger [7,28,95]. Both prenatal and postnatal care tends to be insufficient in MA-addicted mothers. Additionally, these mothers fail to provide the attention needed by their children and essential facilities are often missing [96]. These factors make the direct effects of the drug less distinguishable from the negative external influences, with all the aforementioned aspects having a negative impact on the child’s development [30,84,97,98,99,100,101,102].

## 5. Methamphetamine: Prenatal Influence on the Child

Prenatal exposure to MA is a significant and widely discussed issue. Few clinical studies concerning the direct effects on brain development and subsequent behavior changes have been performed [85]. The required length of the observation period with a sufficiently large set of subjects is the main problem and is probably why no study on adults exposed prenatally to MA has been performed yet [23,103]. Furthermore, the negative effects of street drugs during breastfeeding has been demonstrated for most common drugs, such as cocaine, heroin, MA, and marijuana [104,105]. Several studies have been performed on newborn children shortly after birth [106,107,108,109,110], as well as young preschool children [107,111,112].

Previous studies surmised that using MA leads to an increased frequency of heart defects, cleft lips, congenital atresia, and stillbirths [7,18]. These studies were performed as retrospective analyses with numerous limitations and with small datasets. Newer studies have not found any differences between children with prenatal exposure to MA and controls [113]. Moreover, no abstinence syndrome requiring a medical intervention was observed in newborn children [109]. Available data from developmental studies on infants with prenatal exposure to MA, however, indicate a deceleration of neurobehavioral development manifesting as low-quality movement, decreased excitability, and increased stress levels [85,89,107,114,115]. Newborns exposed to MA often exhibit disorderly, low-quality movement, changes in EEG, and high physical tension [116,117,118]. The aforementioned disorganization, however, tends to disappear during the first month of life [118,119].

Certain studies have described lowered birth length, weight, and head girth, and also an increased occurrence of intrauterine growth retardation [110]. Recent studies tend to confirm these results. Smith et al. [106] described a heightened risk of newborns being small relative to gestational age and lower birth weights. Even after three years, these children were significantly smaller than their peers [120]. Cernerud et al. [121] followed small cohorts of children prenatally exposed to MA from birth to the age of 14. Within the observed group, prenatally exposed girls were still significantly smaller than the control group, even after 14 years.

Fetal growth retardation has been linked to metabolic syndrome and obesity. Slowed growth in newborns can damage their health, which manifests even after reaching adulthood [122]. Other studies offer evidence of its influence on the brain structure [123]. Prenatal exposure to MA leads to a decrease volume of subcortical structures, namely the putamen, globus pallidus, caudate nucleus, and hippocampus [124]. Children prenatally exposed to MA generally have a smaller striatum containing fewer D2 dopamine receptors, along with a general decrease in creatinine levels [32,116]. These children, however, do not exhibit lowered IQs, nor do they suffer from language problems [119,125,126].

Other studies suggest possible issues regarding younger school-aged children related to social integration, getting along with peers, as well as having lower results in cognitive testing [127,128]. Their behavior is often associated with depression, anxiety states, and emotional instability [79,129,130], which manifest as increased externalization and personality disorders [69,131], such as attention deficit hyperactivity disorder (ADHD) [129,132,133,134,135]. Around the age of 7, behavior disorders, often involving aggressive behavior, are commonly diagnosed in these children [111,136]. The strong prenatal effect of MA, together with general “hardship” during childhood, are associated with impaired neurological development [114,130,137].

Currently, only limited data are available regarding the genetic mechanisms participating in the onset of psychiatric disorders or the transference of drug addiction from mother to child [138]. Clinical trials are mostly focused on statistical comparisons of these issues; moreover, it is extremely difficult to follow a sufficiently large group of children prenatally exposed to MA until adulthood [85]. Due to the limited options regarding clinical trials, most prenatal exposure studies are performed using animal models [101,114].

### Tests of MA Prenatal Influence on Offspring

**Figure 3:**
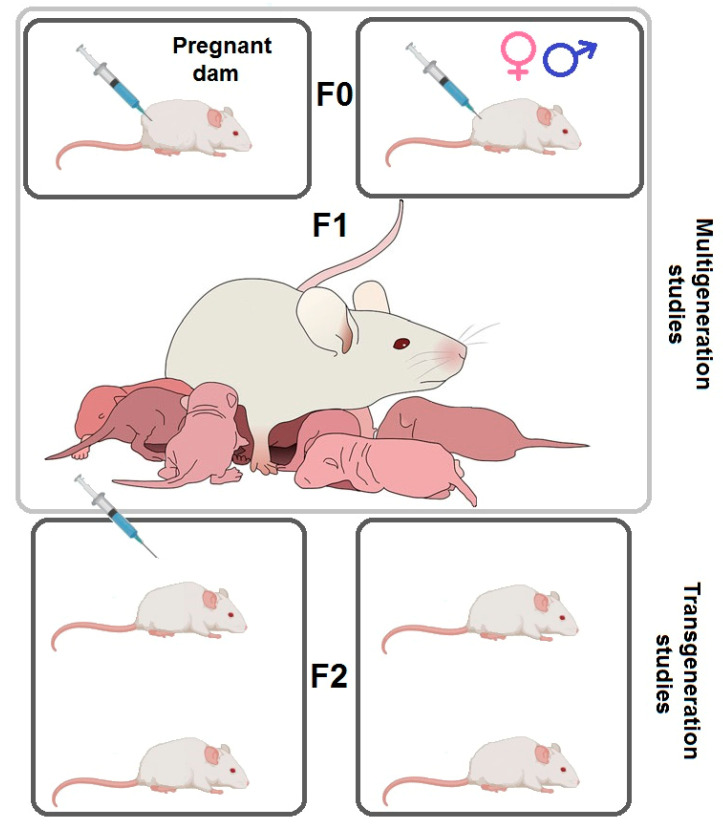
Scheme for testing intergenerational transmission of drug addiction. For the study of multigeneration transmission, the influence of the F0 generation, either with pregnant females only or both male and female rats, is used, then the F1 generation is tested. For the study of transgenerational transmission, the F0 generation is influenced as in the previous case. The F1 generation may or may not be affected and the F2 generation rats are tested.

The prenatal influence of addictive substance use can be studied in several ways. First, multigenerational influence testing, where the drug is administered to the F0 generation (parent), and its impacts are measured in the F1 generation (offspring). In such cases, either both parents or just the pregnant mother can be targeted. Second, transgenerational influence testing, where the drug is administered in either the F0 or both the F0 and F1 generations, and its impacts are measured in the F2 generation. Again, either both parents or just the pregnant mothers can be targeted (Figure 3) [139,140].

At first glance, the differences in development between humans and rats are significant, considering the stark contrast in the length of both prenatal and postnatal development. While humans are born after a nine-month (270 days) pregnancy and reach adulthood after approximately 20 years, total development in rats is finished after approximately six weeks, i.e., the three weeks prior to birth (embryonic day (ED) 0 to 21), and the three weeks after birth (postnatal day (PD) 0 to 21). Both species are relatively immature at the time of birth when considering motor skills. A rat is barely able to raise its head above the supporting surface on day P2 [141,142], which roughly corresponds to the third month in children. By day P5, a rat can life its shoulders off the ground, which is directly connected to the functional maturing of the upper limbs starting to support the upper body weight [142]. In rats, by the end of the first postnatal week, the lower limbs are still not yet capable of supporting the weight of pelvis. Using the limbs for support is first observed on day P10, with the ability to walk appearing on day P12 [141,142]. In comparison, babies usually start walking around the end of their first postnatal year (Table 1) [143].

However, noticeable similarities can be observed during brain development and motor skills when applied to a time axis where one day of rat development corresponds to one month of human development (Table 2) [114,143,145]. We can correlate the time of development of specific neurological structures of the brain, making it possible to track the growth and maturation process.

In rats, we can follow the growth process in the first half of pregnancy. Maturation occurs in the second half of pregnancy and in the early neonatal period. The human brain grows through the second and third trimester and ends around the 40th week of pregnancy. However, maturation and organization continue long after birth. One study suggests that brain development in rats is most vulnerable to MA during day 12–21 prenatally and day 1–11 postnatally, which correlate with the second and third trimesters in humans [114].

In order to fully understand the influence of MA on the CNS, it is necessary to comprehend the development of this system. The nervous system develops and matures from the prenatal period to adulthood; the sequence of corresponding events is generally comparable among different species; however, the time spans can differ significantly [144,146]. The sensibility to a teratogenic agent is variable depending on the development stage of the embryo at the time of exposure. The stage when disruption of organ development by the external agent is possible can be considered the most critical, since in later stages the organ can no longer be structurally damaged. The critical stage is determined by the organ’s morphogenesis period [147,148]. It is also necessary for the critical stage to overlap with the period of sensitivity, i.e., where the cells are sensitive to the agent. Various clinical disorders (schizophrenia, dyslexia, epilepsy, autism) may be the result of an interference with the development of the nervous system. Prenatal exposure to various teratogenic agents (X-rays, methylazoxymethanol, ethanol, lead, methylmercury, chlorpyrifos) in both humans and animals has shown that the exposure of one or more agents can cause neurotoxicity. Different behavioral domains (sensory, motor, various cognitive functions) are dependent on various areas of the brain [51,141,149]). Despite significant differences between human and rat brains, analogous structures can be identified. The ontogenesis of specific behavior can be used to reach conclusions regarding the maturation of specific brain structures or neural pathways in rats, primates, and humans [146,147,148]. The maturation of the most important anatomy structures progress in the same order in both rat and human brains [39].

The animal model studies indicate that using MA in the first and third trimester may have long-term effects on the dopamine and serotonin systems, which could influence learning and social development of the brain [150]. For the rat model, the critical period of organogenesis (within its 20-day pregnancy) occurs between days 8 and 15 [51,151]. Previous studies have shown that MA affects the CNS by increasing catecholamine levels in the cytosol of neurons [42,152]. The increase in catecholamines is caused by reduced amounts of dopamine and NA in the synapses. Their reactive metabolites produce free radicals, inflicting oxidative damage to DNA and leading to cell apoptosis [153]. This process was demonstrated in pregnant female rats that were administered doses of MA at 20 and 40 mg/kg, leading to oxidative damage to DNA and influencing embryonic brain development [32,51,85]. It was also determined that lower MA doses (2–5 mg/kg) during pregnancy cause a decrease in dopamine and serotonin absorption, while also decreasing the binding of serotonin to serotoninergic receptors in the offspring. Higher MA doses (10 mg/kg), by contrast, increased the uptake of dopamine and serotonin, with the MA-exposed offspring exhibiting lower activity compared to controls [51]. Further studies have shown that administering MA (5 mg/kg) during pregnancy affects the sensory motor development of the offspring during the weaning period [154,155,156], affects learning and memory [157], increases susceptibility to seizures [99], and changes pain sensitivity in a gender-specific manner [158].

## 6. Epigenetics of Methamphetamine-Induced Changes

It is currently accepted that epigenetic regulations of CNS occur in the absence of modifications of the DNA sequence itself. This seems to be the set of mitotic changes in gene transcription and phenotypic alterations [159]. Dynamic epigenetic remodeling allows constant variation in the gene readout within cells, and within the CNS all of this can change the neuronal function itself. Epigenetic modifications of our genome are a reflection of the integration genotype environment. The mechanisms include methylation of DNA (especially cytosine methylation), covalent post-translational histone modifications (preferably characterized by acetylation and methylation), and RNA interference (short and long RNAs) [159,160,161].

Current developmental models suggest that an unfavorable environmental, psychosocial experiences, or physical experiences during early life are predisposing factors for the development of behavioral, emotional, and other adulthood disorders. Unstable and unorganized maternal care and a stressful environment adversely affect appropriate behavioral responses and cause maladaptive behavior [162]. It is known that the offspring of people with such behavioral changes (and sometimes generations of these offspring) are often similarly affected, although they do not have trauma [163,164]. Early stress changes the DNA methylation of several candidate genes in the germline of males subjected to maternal separation, as well as in the brain and some germline progeny genes. This suggests that early stress constantly changes behavior and modifies the epigenetic profile of genes over generations [162,163,164]. Transgenerational transmission of complex behavioral changes induced by early stress can also be modelled in animals by an experimental paradigm for chronic and unpredictable stress in early life. The persistent and unpredictable segregation of the mother during early postnatal development in mice and rats causes depressive behavior and changes the response of animals to a new and resistance environment [84,163,165,166]. Most observed behavioral changes passed to the offspring of males who were separate from their mother [84]; however, those offspring also pass this behavior on to the next generation [84,166].

Drug usage in pregnancy affects two developmental periods in which epigenetic reprogramming of the genome occurs. It affects the germ cells and preimplantation embryos. According to Itzhak [167], prenatal exposure to MA causes changes in offspring behavior and DNA methylation patterns in the hippocampus. In the embryonic period, epigenotype and behavioral phenotypes of progeny vulnerable to MA exposure occurs. Extensive reprogramming of epigenetics takes place during this period. These changes can be modulated postnatally. Both DNA methylation and adolescent behavior modulate maternal behavior. As such, MA exposure before and during pregnancy has been shown to result in significant persistence effects on DNA methylation, which may subsequently affect gene expression and cause abnormal phenotypes throughout life [167,168].

Itzhak [167] revealed altered behavior in the offspring of the F1 generation. This may be a direct consequence of the action of MA on germ cells in the uterus, an indirect result of altered maternal behavior, or their combination. Although the examination of maternal behavior did not reveal significant differences between MA and normal offspring concerning breastfeeding, self-concept, and time spent outside pups, the results of cross-fostering studies suggest that some behavioral phenotypes of F1 offspring were affected by maternal care [23,84,99]. The methylation of a large number of gene promoters in the F1 generation resulting from the use of MA by a pregnant mother was altered, enhanced, or reduced, depending on the mother’s postnatal care. This finding suggests that maternal behavior and MA contributes significantly to the offspring’s epigenetic response [167]. Epigenetic phenomena are involved in the clinical manifestations of neuropsychiatric diseases, which also include addiction [169]. MA engenders transcriptional and epigenetic changes that are unique to this [170]. MA can induce epigenetic modifications that underlie persistent changes in gene expression and long-lasting behavioral responses to the drug [159]. DNA hypomethylation occurs in promoters of genes related to DA metabolism. It is present in the gene promoter of DRD3, DRD4, and membrane-bound catechol-O-methyltransferase (MB-COMT) genes. COMT provides methylation of a hydroxyl group of DA-forming 3-methoxytyramine. In psychotic MA, users experience hypomethylation of the MB-COMT gene promoter and increased expression of COMT associated with synaptic DA degradation in the prefrontal cortex [171,172]. Additionally, DNA hypomethylation of the AKT1 promoter gene is detected [171]. The AKT1 gene encodes a serine–threonine kinase protein, which is mostly expressed in the brain and linked to DNA transcription, neural survival and growth, synaptic plasticity, and working memory [173,174].

Differentially methylated regions in promoters of genes are associated with the response to MA, such as Adora1, Camkk2 [175], Hdac5 [176], and Camk2a. Calcium-dependent kinases (CamKK2 and Camk2a) and histone deacetylase 5 (Hdac5) are involved in synaptic plasticity, learning, and memory. Therefore, differentially methylated regions in the promoters of these genes results in changes of the behavioral phenotype, which are associated with appetitive and aversive associative learning [167].

The effects of MA on memory are evident, such as in rats with Parkinson’s-like behavior. Parkinson’s disease and Parkinson’s-like behavior have been studied most in postmortem brains and rodents. Tan et al. [177] reported significant hypomethylation in the CpG sites in the promoter region of α-synuclein (Snca) in the leukocytes and postmortem brain samples. Decreases of nuclear DNA methyltransferase 1 levels, which lead to DNA hypomethylation in the CpG islands upstream of Snca, were observed in postmortem brain samples from the patients with Parkinson’s disease or dementia [178]. The exposure to MA decreased cytosine methylation in the Snca promoter region and in the substantia nigra, which led to upregulation of α-synuclein. The epigenetic upregulation of α-synuclein potentially contributed to the Parkinson’s-like behavior in the rodents with previous use of MA [170,179].

There is a suggestion that the epigenetic effects of MA in the brain might depend on the pattern of MA use. Chronic MA abuse decreased striatal messenger RNA (mRNA) expression of ionotropic glutamate receptors (GluA1 and GluA2) and also caused decreased GluA1 and GluA2 protein levels. According to Jayanthi et al. [180,181,182], MA caused a significantly decreased abundance of striatal histones (H4K5ac, H4K12ac, and H4K16ac). Chronic use is different from the single injection of MA (20 mg/kg) that caused a time-dependent increased abundance of H4K5ac and H4K8ac in the nucleus accumbens [183].

The role of epigenetics in the MA influence on the brain is quite obvious, and we need deeper insight to understand the molecular mechanisms of addiction. This is key, since MA possesses a variety of effects occurring in some neuropsychiatric disorders. As we are constantly uncovering novel epigenetic changes, it is clearer how often small transient changes in the balance of neurotransmitters are reflected in permanent changes in brain physiology.

## 7. Conclusions

The long-term effects on the organism always seem less significant in comparison to measurements performed immediately after birth. Males showed lower susceptibility to prenatal drug influence than females according to several studies [98,139,184,185,186,187]. Exposure to stress is another important factor that can significantly intensify the effects of MA [188]. Women addicted to MA are often unemployed (85%) [28], with both prenatal and postnatal care often being inadequate, i.e., mothers do not attend to their children’s needs and necessary facilities are often missing as well. Therefore, any direct impacts of the drug are difficult to distinguish from external negative factors; however, all factors collectively have a negative impact on child development [30,98,99,102,189]. It is also assumed that MA is capable of strongly augmenting the effects of stress. The drug dose is an important issue as well.

Previous studies have shown that the range of dosage of 5–10 mg/kg reaches concentrations in the rat embryonic brain similar to those reached in the brain of children of addicted women [155]. Others have used a single dose of 5 mg/kg in their studies [84,97,98,187]. Such a dose does not shorten the gestation period and does not cause miscarriages or embryo malformations. This aspect, however, means that the correlation to clinical data is problematic, since clinical studies showed that at the time of conception, mothers were already addicted to the drug for an average of three years. In rat studies, however, drug administration is different from the drug abuse pattern of addicted women. Pregnant women tend to increase their dosages during pregnancy, starting with significantly lower doses compared to the end of the pregnancy, while in rat studies the pregnant females received consistent doses of 5, 10, 15, or 20 mg/kg during the entire period [155]. Therefore, females in rat studies can be considered to have received the average dose taken by pregnant woman.

Furthermore, comparing doses administered to rats by a researcher or through self-administration, the increase in the amount of drug taken over time is noticeable. During self-administration, a single dose is around 0.05 mg/kg over the course of the experiment; however, the rat administers up to 8–9 mg/kg every six hours (so-called progressive ratio schedule). Rats administer the dose several times a day, with dosages of up to 25–48 mg/kg/day, with apparent differences between sexes [190,191].

In this context, it is important to consider the fact that most studies concerning the effects of MA are performed either on animal models or by evaluating the effects of MA use in addicted individuals. The animal model studies enable the administration of pharmacologically controlled doses and numerous histological processes that cannot be performed on human subjects for obvious ethical reasons. However, most animal studies do not allow us to reach definite conclusions concerning the processes taking place in humans due to differences in pharmacokinetics, cell function, dosage, and behavior. Studies on humans allow us to reach these conclusions, however it is impossible to control all of the variables and the results are often distorted by comorbidity or polysubstance abuse.

Considering the advantages and disadvantages of both lines of research, it would be beneficial to link animal and human studies more closely by aligning the procedures. This would enable the same (or at least corresponding) neurobiochemical, neurophysiological, electrophysiological, behavioral, and cognitive processes in both human and animal subjects to be studied in a way that would partially overcome the limitations of both models and provide a more causal, mechanistic view of the effects and consequences of MA toxicity. Tight coordination would be required in order to gain all of the benefits of this approach, such as achieving a deeper understanding of the issue and further integration of the findings from both areas of research [192,193]. Special attention should be paid to research into neurobiological and neurophysiological mechanisms, since these represent the origins of potential cognitive process dysfunctions affecting a child’s school readiness and success in school and work life, as much as their physical and psychological health [194]. Apart from the direct effects of the drug on the child and the detrimental effects of MA persisting in society into the future, it is necessary to consider the indirect effects as well. In order to fully understand and accurately determine the significance of the possible consequences of both multi- and transgenerational MA addiction transfer, it is essential to commence such cognitive and neurological research as soon as possible.

## Figures and Tables

**Table 1:** Comparison of the length of developmental periods of rats and humans. The experimental rat lives up to about 3 years and humans average about 80 years. Calculation: (80 × 365) ÷ (3 × 365) = 26.7 human days corresponds to one day in rat life. At the same time: 365 ÷ 26.7 = 13.8 days in the life of a rat corresponds to one year of human life. Adapted and edited by Sengupta [144].

Life Period	Rat	Man	Influence of MA
Life expectancy	2–3 years	75–85 years	Highest addiction in the 15–34 age range [2,3,4]
Weaning	week 3–4	after 6–12 months	MA passes into the milk [88,105]
Prepubertal period	4th week	Between 8–10 years	The influence of environment on the psycho-motoric development of the child [31,33,87,106,111,112,116,128,129,133,135]
Adolescence	week 5–10	10–20 years	First use of addictive substance [2,24,133,137]
Adulthood	From week 10 to 12	From year 20	Period of sexual maturity—pregnant addicted woman [2,7,18,23,31,135]
Late age	From 20th month	From the 60th year	

**Table 2:** Comparison of pre- and perinatal development of the brain in rats and humans. The influence of MA on the brain development (in the sense of slowing development) is highlighted in the table. Adapted and edited by Šlamberová [114]; Petríková and Šlamberová [145]. ED—embryotic day.

Brain Development	Rat	Man
Neurulation and development of neural tube	9–11 ED (half of pregnancy)	24–28 ED (3rd week of pregnancy)
Cell proliferating process	10.5–11 ED	From 4th week of pregnancy
Serotoninergic cells	9–11 ED	5th week of pregnancy
Striatum, nucleus accumbens, basal ganglia	12–13 ED	5th week of pregnancy
Noradrenergic neurons	12–14 ED	5th–6th week of pregnancy
Dopaminergic neurons	10–15 ED	6th–8th week of pregnancy
Hippocampus, amygdala, limbic system	From 14 ED (about 15% of granular cells are created in the postnatal period)	8th week of pregnancy (about 80% of granular cells are created in the 40th week, i.e., right before labor)
Maturation of synaptic connections	18–21 ED (last prenatal days)	34th–36th week of pregnancy
The peak of neurogenesis	1st and 2nd week postnatal	40th week of pregnancy
